# Pterostilbene downregulates BCR/ABL and induces apoptosis of T315I-mutated BCR/ABL-positive leukemic cells

**DOI:** 10.1038/s41598-021-04654-1

**Published:** 2022-01-13

**Authors:** Shohei Kawakami, Mitsuyo Tsuma-Kaneko, Masakazu Sawanobori, Tomoko Uno, Yoshihiko Nakamura, Hideyuki Matsuzawa, Rikio Suzuki, Makoto Onizuka, Takashi Yahata, Kazuhito Naka, Kiyoshi Ando, Hiroshi Kawada

**Affiliations:** 1 Division of Hematology/Oncology, Department of Internal Medicine, Tokai University School of Medicine, 143 Shimokasuya, Isehara, Kanagawa 259-1143 Japan; 2 Research Center for Regenerative Medicine, Tokai University School of Medicine, 143 Shimokasuya, Isehara, Kanagawa 259-1143 Japan; 3 Support Center for Medical Research and Education, Tokai University School of Medicine, 143 Shimokasuya, Isehara, Kanagawa 259-1143 Japan; 4 Department of Innovative Medical Science, Tokai University School of Medicine, Tokai University School of Medicine, 143 Shimokasuya, Isehara, Kanagawa 259-1143 Japan; 5 Department of Stem Cell Biology, Research Institute for Radiation Biology and Medicine, Hiroshima University, 1-2-3 Kasumi, Minami-ku, Hiroshima, 734-8553 Japan

**Keywords:** Cancer, Medical research, Oncology

## Abstract

In this study, we examined the antileukemic effects of pterostilbene, a natural methylated polyphenol analog of resveratrol that is predominantly found in berries and nuts, using various human and murine leukemic cells, as well as bone marrow samples obtained from patients with leukemia. Pterostilbene administration significantly induced apoptosis of leukemic cells, but not of non-malignant hematopoietic stem/progenitor cells. Interestingly, pterostilbene was highly effective in inducing apoptosis of leukemic cells harboring the *BCR/ABL* fusion gene, including ABL tyrosine kinase inhibitor (TKI)-resistant cells with the T315I mutation. In BCR/ABL^+^ leukemic cells, pterostilbene decreased the BCR/ABL fusion protein levels and suppressed AKT and NF-κB activation. We further demonstrated that pterostilbene along with U0126, an inhibitor of the MEK/ERK signaling pathway, synergistically induced apoptosis of BCR/ABL^+^ cells. Our results further suggest that pterostilbene-promoted downregulation of BCR/ABL involves caspase activation triggered by proteasome inhibition-induced endoplasmic reticulum stress. Moreover, oral administration of pterostilbene significantly suppressed tumor growth in mice transplanted with BCR/ABL^+^ leukemic cells. Taken together, these results suggest that pterostilbene may hold potential for the treatment of BCR/ABL^+^ leukemia, in particular for those showing ABL-dependent TKI resistance.

## Introduction

ABL tyrosine kinase inhibitors (TKIs) have significantly improved the prognosis of patients with chronic myeloid leukemia (CML) and acute lymphoblastic leukemia (ALL)^1–3^. A large proportion of CML patients attain a prolonged molecular response, and in approximately half of the patients who are treated with ABL TKIs and achieve a certain period of sustained deep molecular response, ABL TKI administration can be discontinued and treatment-free remission can be achieved^1^. However, the remaining patients require continued therapy to prevent relapse and consequently experience problems due to long-term treatment, such as adverse outcomes and high treatment cost^2,4^. Furthermore, many CML and ALL patients have shown multi-TKI failure^1,5^. In particular, in patients harboring the T315I *ABL* mutation, which is the most frequently observed mutation among all compound mutants, administration of first-generation (imatinib) and second-generation (dasatinib, nilotinib, and bosutinib) ABL TKIs failed to prevent BCR/ABL expression; although administration of the third-generation ABL TKI ponatinib is still a possibility, its toxicity profile is often problematic^1,5^. Thus, the development of new therapeutic strategies for BCR/ABL^+^ leukemias is still important.

Dietary stilbenes comprise a class of natural compounds that display significant biological activities of medicinal interest^6^. Resveratrol is a well-known natural phytoalexin produced by a wide variety of plants, such as grapes, blueberries, and nuts, in response to environmental stress^6^, which has attracted significant attention in several fields of human health, as confirmed by the number of publications concerning its antioxidative and anti-inflammatory activities. Given its functional activities, resveratrol is also quite popular as a nutritional supplement^7,8^. Recent reports have further demonstrated the antiproliferative and proapoptotic activities of resveratrol against various human cancers, including leukemia^7,9^. However, the low bioavailability of the parental compound, as consequence of its poor resorption and extensive biotransformation, is a limitation for the therapeutic use of this molecule^10^.

Similar to resveratrol, pterostilbene (trans-3,5-dimethoxy-4-hydroxystilbene), a natural dimethyl ether analog of resveratrol, is predominantly found in berries and nuts. From a structural point of view, pterostilbene and resveratrol are quite similar^11,12^; however, pterostilbene has superior oral adsorption and metabolic stability owing to the presence of only one hydroxy group in its structural backbone^6^. Furthermore, pterostilbene has been reported to exhibit anticancer activities via various mechanisms in several common malignant tumors, including hematologic malignancies^13–19^.

Herein, we explored the therapeutic potential of pterostilbene on various hematological malignancies, especially in BCR/ABL^+^ leukemia, and also explored the underlying molecular and cellular mechanism by which pterostilbene exerts its effects on these leukemic cells.

## Results

### Pterostilbene specifically induces apoptosis of BCR/ABL^+^ leukemic cells, including TKI-resistant T315I-mutated BCR/ABL^+^ cells

**Figure 1:**
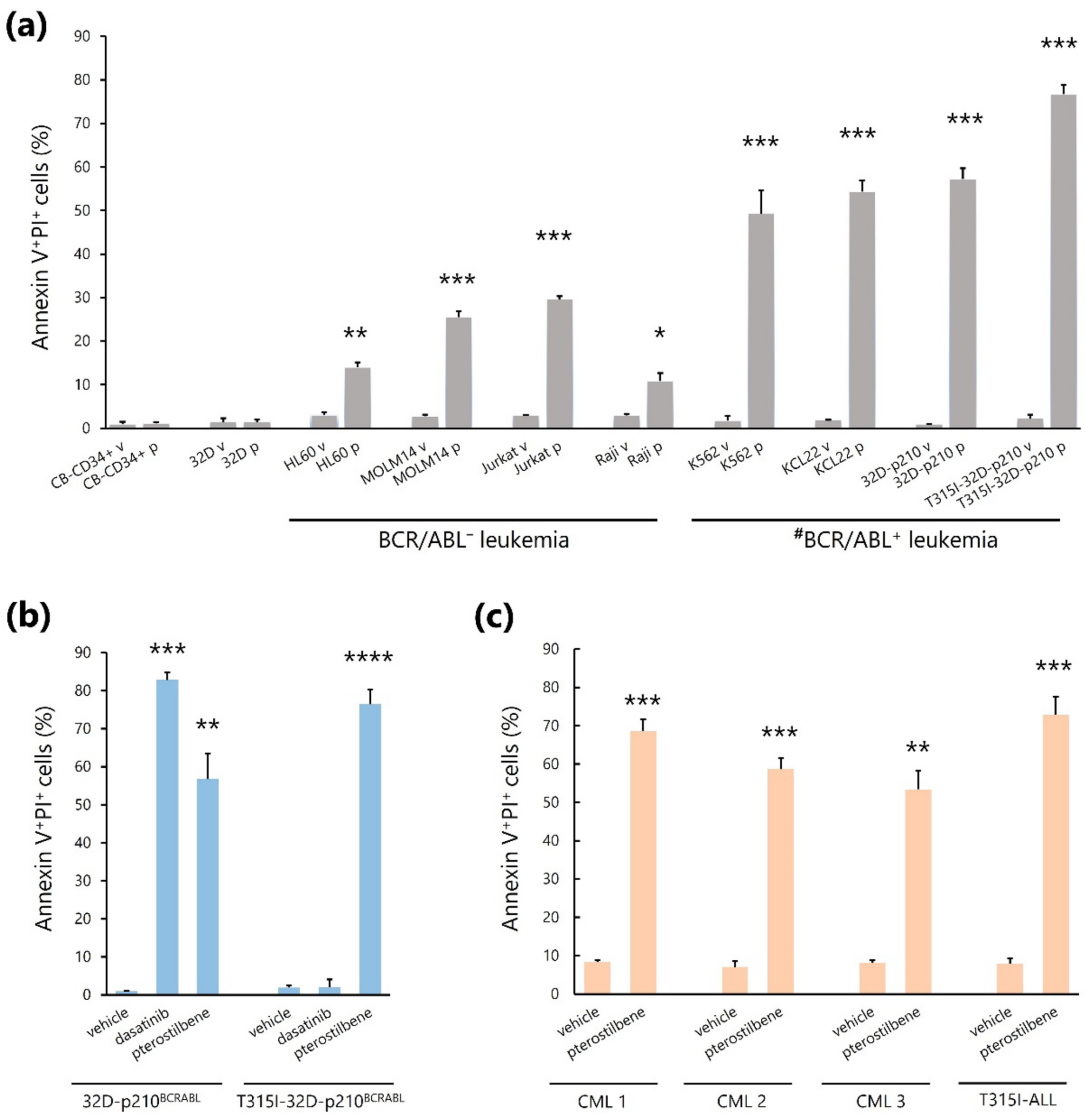
Flow cytometric measurement of apoptosis induced by pterostilbene in various leukemic cell lines, normal hematopoietic progenitor/stem cells, and primary leukemic samples. (**a**) Measurement of cell apoptosis in the presence of a control vehicle (v) or pterostilbene (p). *P < 0.01, **P < 0.001, ***P < 0.0001 compared with the vehicle. Values represent the mean ± SD of triplicate samples. ^**#**^Apoptosis was induced by pterostilbene in a significantly higher percentage of BCR/ABL^+^ leukemic cells than in BCR/ABL^−^ leukemic cells (*P* < 0.01). (**b**) Measurement of cell apoptosis in the presence of vehicle, dasatinib or pterostilbene. **P < 0.001, ***P < 0.0001 compared with the vehicle. ****P < 0.0001 compared with the vehicle or dasatinib. Values represent the mean ± SD of triplicate samples. (**c**) Measurement of cell apoptosis in primary leukemic samples. **P < 0.001, ***P < 0.0001 compared with the vehicle. Values represent the mean ± SD of triplicate samples.

Firstly, we assessed the apoptosis-inducible activity of pterostilbene in vitro. The addition of pterostilbene significantly induced apoptosis of various human leukemic cells, but not of human umbilical cord blood-derived CD34^+^ normal hematopoietic stem/progenitor cells and murine hematopoietic cells (32D cells; Fig. 1a, Supplementary Figs. 1 and 2). Interestingly, pterostilbene strongly induced apoptosis of BCR/ABL^+^ leukemic cells than of BCR/ABL^−^ leukemic cells (Fig. 1a). In contrast to 32D-p210^BCR/ABL^ cells, T315I-32D-p210^BCR/ABL^ cells were resistance to dasatinib; nonetheless, pterostilbene was still able to induce apoptosis of most of these cells (Fig. 1b). We further assessed the apoptosis-inducible effect of pterostilbene using frozen bone marrow mononuclear cells (BMMNCs) isolated from four patients: three with CML in the chronic phase at diagnosis and one with relapsed ALL harboring the T315I *ABL* mutation. Pterostilbene administration also significantly induced apoptosis of BMMNCs from all four patients (Fig. 1c).

### Pterostilbene decreases BCR/ABL levels and inhibits its downstream targets

**Figure 2:**
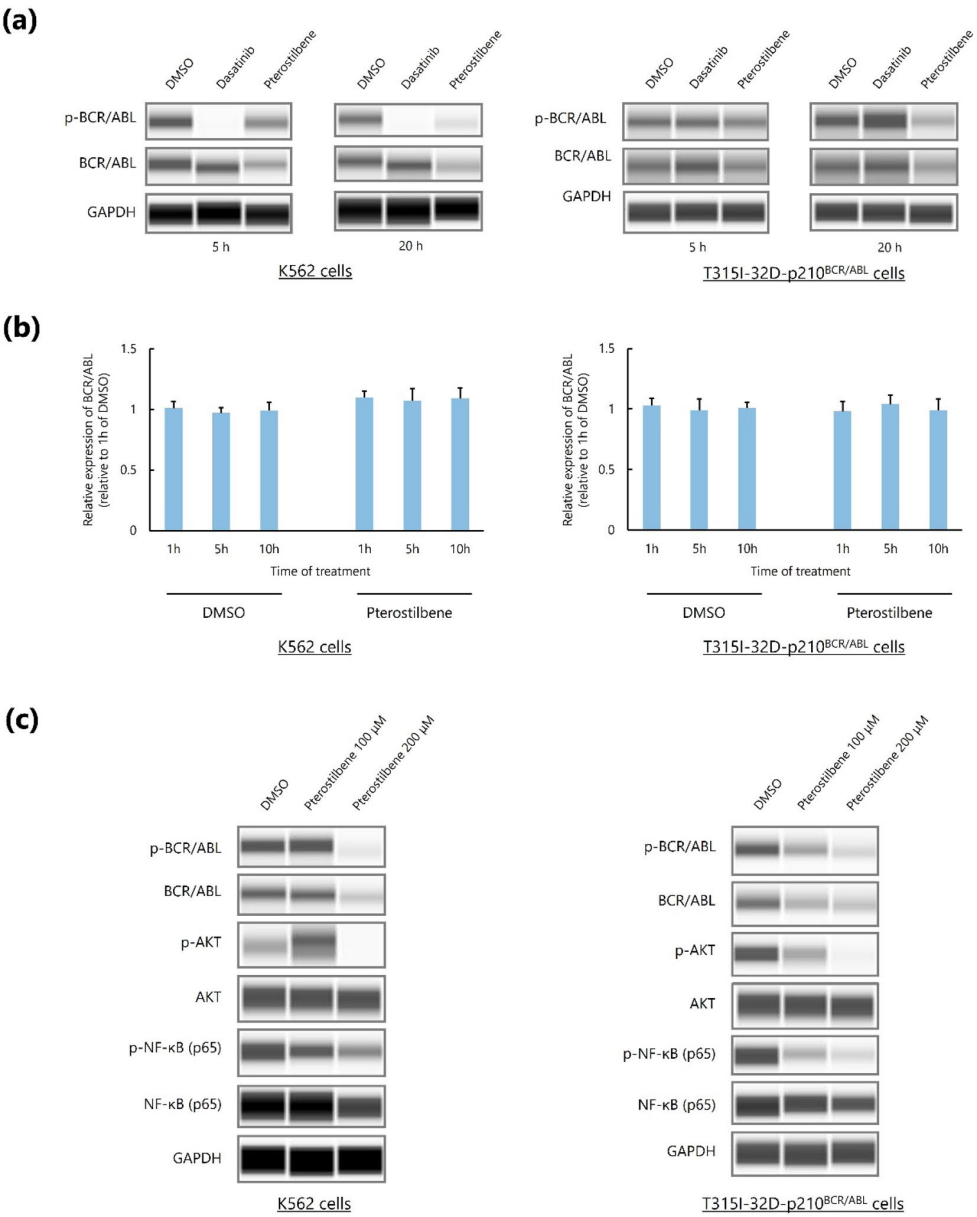
Pterostilbene promotes downregulation of BCR/ABL and its downstream signals in BCR/ABL^+^ leukemic cells. (**a**) Capillary western immunoassay. GAPDH was used as loading control. (**b**) Real-time PCR analysis of *BCR/ABL* expression. Values represent the mean ± SD of triplicate samples. (**c**) Capillary western immunoassay. GAPDH was used as loading control.

Next, we examined the molecular mechanism involved on the apoptotic effect of pterostilbene in BCR/ABL^+^ leukemic cells. Dasatinib administration reduced BCR/ABL phosphorylation levels in K562 cells but not in T315I-32D-p210^BCR/ABL^ cells, whereas pterostilbene administration significantly reduced BCR/ABL and phospho-BCR/ABL levels in both K562 and T315I-32D-p210^BCR/ABL^ cells (Fig. 2a, Supplementary Fig. 3). The mRNA levels of *BCR/ABL* in K562 and T315I-32D-p210^BCR/ABL^ cells were not significantly affected by pterostilbene administration (Fig. 2b). These results suggest that pterostilbene administration decreases BCR/ABL levels at the post-transcriptional level in BCR/ABL^+^ cells. In addition, pterostilbene suppressed the activation of BCR/ABL downstream targets^20,21^, AKT and NF-κB, in BCR/ABL^+^ cells (Fig. 2c, Supplementary Fig. 4).

### Pterostilbene synergizes with a MEK/ERK inhibitor to induce apoptosis of BCR/ABL^+^ cells

**Figure 3:**
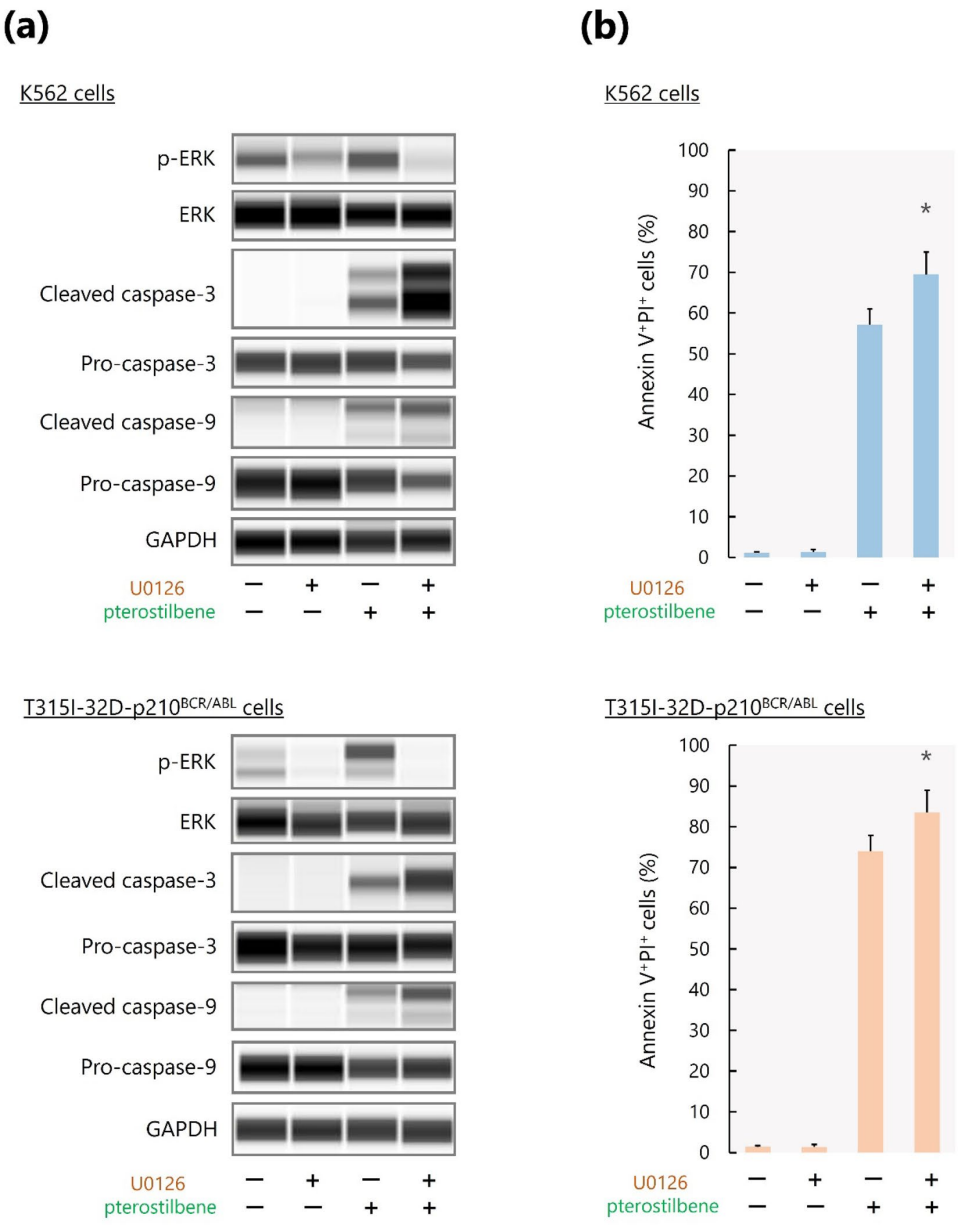
Synergistic effects of pterostilbene and a MEK/ERK inhibitor on BCR/ABL^+^ leukemic cell apoptosis. (**a**) Capillary western immunoassay. GAPDH was used as loading control. (**b**) Measurement of apoptosis by flow cytometry. *P < 0.05 compared with pterostilbene alone. Values represent the mean ± SD of triplicate samples.

We further observed the effect of pterostilbene administration on the MEK/ERK pathway, which is also an important target/effector of BCR/ABL along with AKT/NF-κB and is associated with pro-survival events^22–27^. Interestingly, we found that pterostilbene administration enhanced ERK activation in K562 and T315I-32D-p210^BCR/ABL^ cells (Fig. 3a, Supplementary Fig. 5). In contrast, the MEK inhibitor U0126 inhibited ERK activation but did not enhance caspase activity or induce apoptosis in either cell line (Fig. 3a, Supplementary Fig. 5). However, the combined administration of pterostilbene and U0126 inhibited ERK activation and synergistically induced caspase activation and apoptosis in both cell lines (Fig. 3a,b, Supplementary Fig. 5).

### Pterostilbene-induced BCR/ABL downregulation results from caspase-dependent cleavage

**Figure 4:**
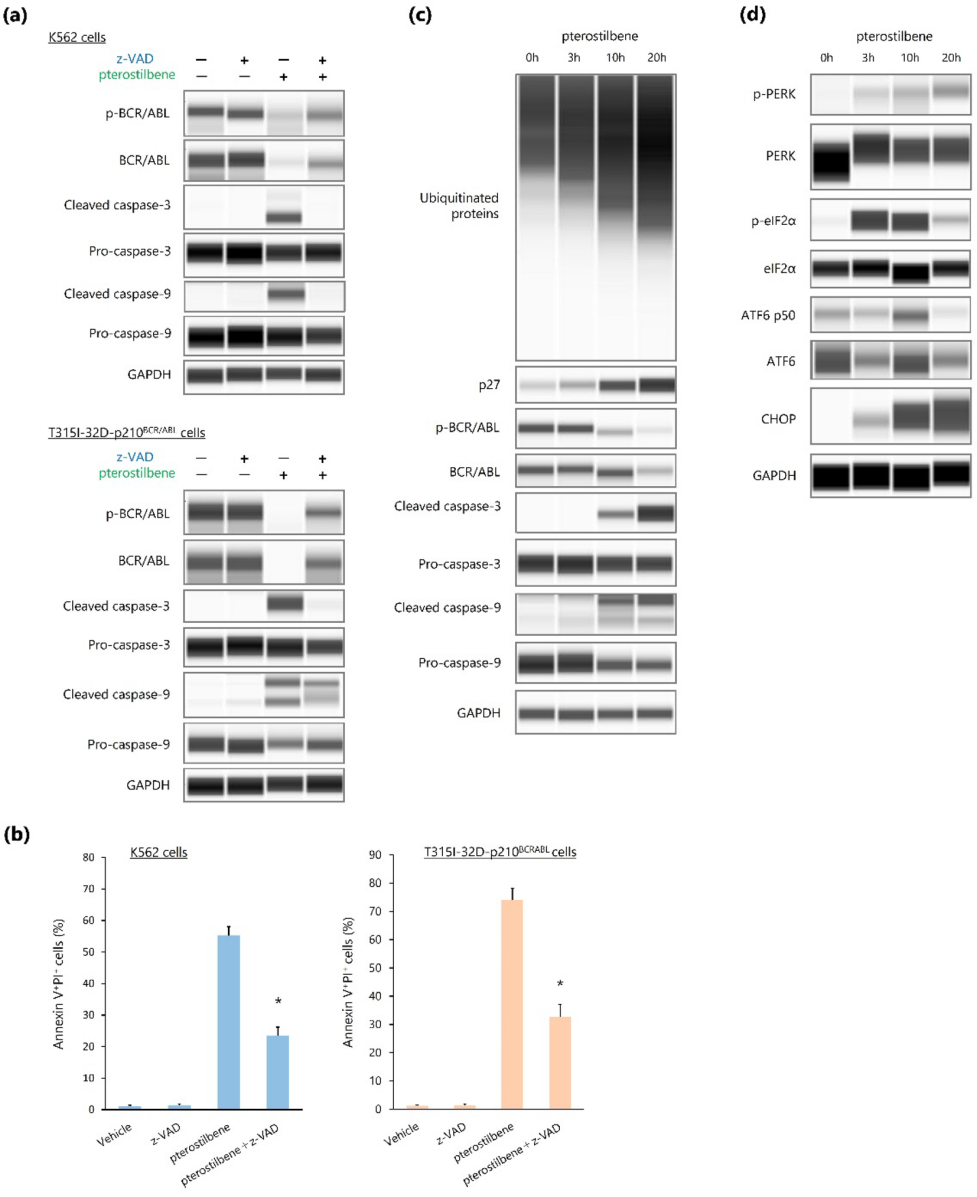
Pterostilbene promotes caspase-dependent downregulation of BCR/ABL. (**a**) Capillary western immunoassay. GAPDH was used as loading control. (**b**) Measurement of apoptosis by flow cytometry. *P < 0.001 compared with pterostilbene. Values represent the mean ± SD of triplicate samples. (**c**,**d**) Capillary western immunoassay. Changes over time when T315I-32D-p210^BCR/ABL^ cells were cultured in the presence of pterostilbene. GAPDH was used as loading control.

Caspase-dependent cleavage is involved in the decrease of BCR/ABL levels in BCR/ABL^+^ leukemic cells triggered by the administration of several antileukemic drugs^26,28–32^; thus, we examined whether caspase activation could also be associated with the observed pterostilbene-induced BCR/ABL downregulation. We incubated K562 and T315I-32D-p210^BCR/ABL^ cells with pterostilbene in the absence or presence of a pan-caspase inhibitor, z-VAD. Pterostilbene administration induced the activation of caspase-3 and caspase-9, and reduced BCR/ABL levels in both cell lines; however, presence of z-VAD inhibited these events, suggesting that BCR/ABL downregulation due to pterostilbene administration primarily reflects caspase-mediated protein degradation (Fig. 4a, Supplementary Fig. 6). We investigated further the effect of z-VAD on the apoptotic effect of pterostilbene in BCR/ABL^+^ cells and found that addition of z-VAD markedly, but not completely, inhibited the pterostilbene-induced apoptosis (Fig. 4b).

While many studies have recently found that the ubiquitin–proteasome system plays a key role in endoplasmic reticulum (ER) stress^33^, caspase activation caused by proteasome inhibition-induced ER stress has been suggested by several reports to contribute to BCR/ABL downregulation^28,32^. Therefore, we investigated whether pterostilbene could have a proteasome-inhibitory effect. When T315I-32D-p210^BCR/ABL^ cells were treated with pterostilbene, ubiquitinated protein levels increased over time, suggesting proteasome inhibition, followed by the activation of caspases and downregulation of BCR/ABL (Fig. 4c, Supplementary Fig. 7). Furthermore, in T315I-32D-p210^BCR/ABL^ cells treated with pterostilbene, ER stress sensors^34^, such as the protein kinase R-like endoplasmic reticulum kinase (PERK) and the activating transcription factor 6 (ATF6), were activated (Fig. 4d, Supplementary Fig. 8). In addition, pterostilbene treatment also promoted the expression of the CCAAT/enhancer binding protein homologous protein (CHOP; Fig. 4d, Supplementary Fig. 8), which was induced by both the PERK/eIF2a signaling pathway and the cleaved activated form of ATF6 (ATFp50), and consequently activated the caspase cascade^34–36^. Together, these results suggest that the downregulation of BCR/ABL due to pterostilbene administration involves caspase activation downstream of proteasome inhibition-induced ER stress.

### Pterostilbene administration effectively represses the proliferation of BCR/ABL^+^ leukemic cells in vivo

**Figure 5:**
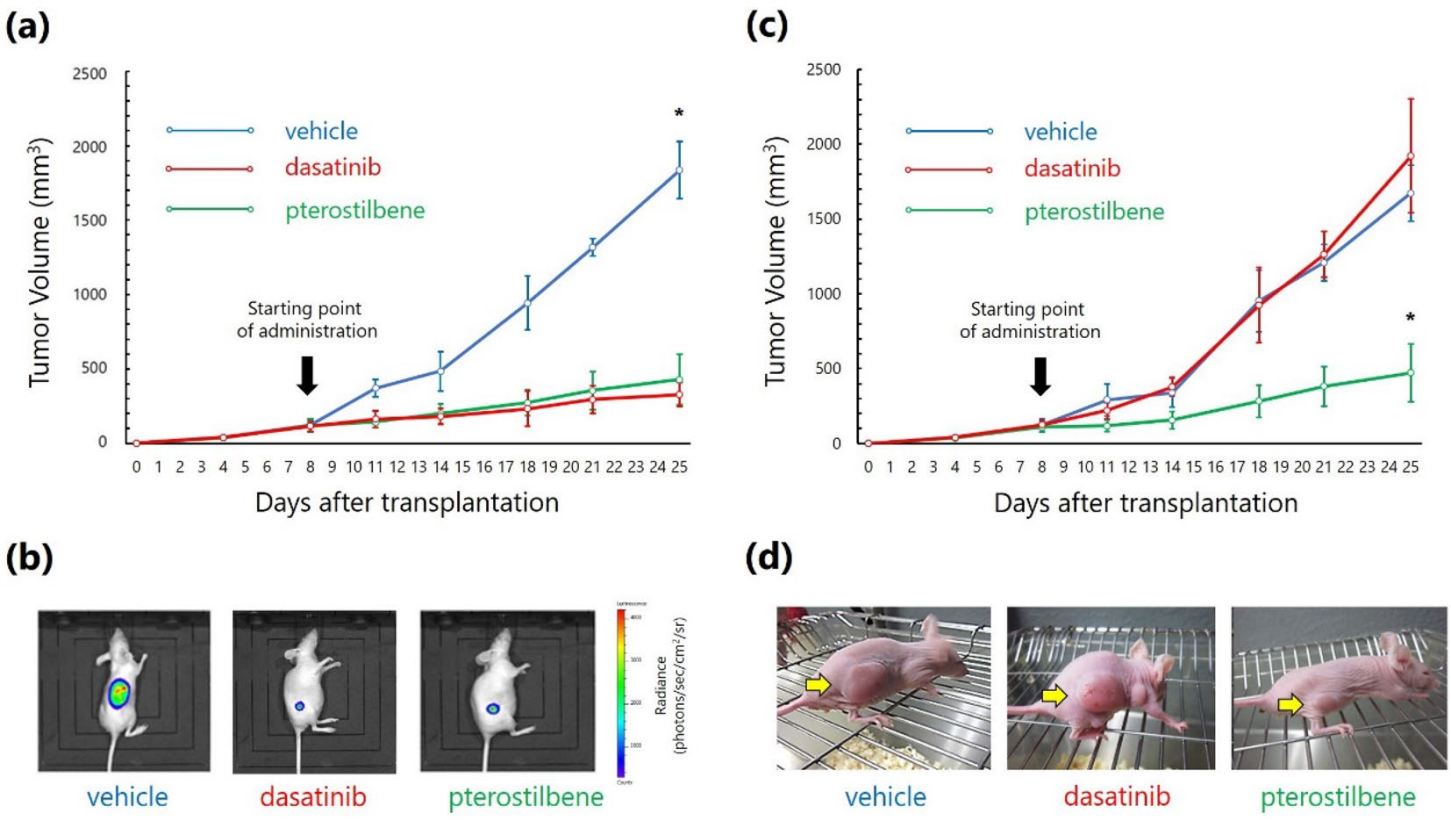
In vivo effects of pterostilbene administration on the proliferation of BCR/ABL^+^ leukemic cells. (**a**,**b**) Mice were transplanted with Luc-K562 cells and treated with vehicle, dasatinib, or pterostilbene. (**a**) Measurement of tumor volume. *P < 0.0001 compared with dasatinib- or pterostilbene-treated mice on day 25 after transplantation. Values represent the mean ± SD of the measurements of five mice. (**b**) Bioluminescence imaging of Luc-K562 cells in mice 25 days after transplantation. Representative images of each group are shown. (**c**,**d**) Mice were transplanted with T315I-32D-p210^BCR/ABL^ cells and treated with vehicle, dasatinib, or pterostilbene. (**c**) Measurement of tumor volume. *P < 0.0001 compared with vehicle- or dasatinib-treated mice on day 25 after transplantation. Values represent the mean ± SD values of the measurements of five mice. (**d**) Appearance of the mice on day 25 after transplantation. Representative images of each group are shown.

Lastly, we examined the effect of pterostilbene administration on the proliferation of K562 and T315I-32D-p210^BCR/ABL^ leukemic cells in vivo. We transplanted the leukemic cells subcutaneously into the right flank of immunodeficient mice and orally administered the investigational compounds. In mice transplanted with K562 cells that stably expressed luciferase (Luc-K562 cells), pterostilbene and dasatinib administration significantly inhibited tumor growth as compared with in the vehicle control (Fig. 5a,b). In mice transplanted with T315I-32D-p210^BCR/ABL^ cells, although dasatinib administration exhibited no inhibitory effect on tumor growth, pterostilbene administration significantly prevented tumor growth (Fig. 5c,d). General toxicities of the reagents were also measured, and no noticeable behavioral changes or morbid consumption, such as significant weight loss or death, were observed (data not shown).

## Discussion

Phytochemicals are natural compounds from plants that represent vital resources for cancer treatment^37–41^, including taxol analogs, vinca alkaloids, and podophyllotoxin analogs^41^. These phytochemicals have significant antitumor potential, but adverse events due to toxicity are often a challenge. In the present study, we found that pterostilbene, a natural polyphenolic compound and a methylated analog of resveratrol, significantly induces apoptosis in BCR/ABL^+^ cells, including of dasatinib-resistant cells harboring the T315I *ABL* mutation. In most in vitro experiments, we used 200 μM pterostilbene, which is a higher concentration than that used in previous reports in which the antileukemic effect of pterostilbene was observed (~ 150 μM)^15–18^; nonetheless, no apparent negative effect was herein observed on normal hematopoietic stem/progenitor cells regarding their survival. We have also confirmed that even lower concentrations of pterostilbene can effectively induced apoptosis of BCR/ABL^+^ cells with longer incubation time (Supplementary Fig. 2).

Pterostilbene was also shown to significantly reduced the levels of BCR/ABL and inhibited its downstream targets AKT/NF-κ-B. However, pterostilbene administration enhanced the activation of ERK, which is a critical target/effector of BCR/ABL and is associated with antiapoptotic actions^22–27^. These findings agree with a previous study that showed that downregulation of BCR/ABL activates MEK/ERK via prosurvival growth factor-mediated signals through a BCR/ABL-mediated negative feedback^27^. Importantly, pterostilbene along with a MEK inhibitor were shown to synergistically enhance caspase activation and consequently trigger apoptosis of BCR/ABL^+^ leukemic cells.

Our results suggest that pterostilbene-promoted downregulation of BCR/ABL involves caspase activation triggered by proteasome inhibition-induced ER stress. It has been reported that BCR/ABL is associated with an increase in proteasome activity^42^ and that proteasome activity is higher in BMMNCs from patients with CML than in healthy controls^43^, suggesting that CML cells may be more susceptible to proteasome inhibition. In fact, it has been reported that BCR/ABL^+^ cells are more sensitive than BCR/ABL^-^ cells to induced apoptosis by proteasome inhibitors^42,44^. It was also suggested that inhibition of the proteasome to trigger ER stress could be an alternative strategy for treating CML^44^. Therefore, we speculate that the mechanism by which pterostilbene strongly induces BCR/ABL^+^ cells to undergo apoptosis is also largely due to caspase activation as a consequence of ER stress caused by proteasome inhibition. Indeed, in this study, the pan-caspase inhibitor z-VAD significantly, but not completely, prevented the pro-apoptotic effects of pterostilbene, but it also induced apoptosis in BCR/ABL^−^ leukemic cells, suggesting that other mechanisms are involved in the pterostilbene downstream effects, as previously reported^15–19^.

Although many studies have reported that resveratrol exerts antileukemic effects^45^, opposite results were obtained in vivo, which may be due to its low bioavailability and because it mainly exists in vivo as glucuronide-conjugated and sulfated metabolites^46–48^. In contrast, pterostilbene exhibits superior oral adsorption and metabolic stability compared with resveratrol^6,10^. Herein, pterostilbene was orally administered to mice transplanted with K562 or T315I-mutated BCR/ABL^+^ leukemic cells. We found that pterostilbene administration significantly inhibited leukemic cell proliferation at a dose that did not cause general toxicity in vivo. Oral administration of pterostilbene has also been reported to be safe and well-tolerated in randomized, double-blind, placebo-controlled studies in healthy subjects or patients with hypercholesterolemia^49,50^.

Collectively, pterostilbene administration may be a novel noninvasive treatment option for patients with BCR/ABL^+^ leukemia, especially for those resistant or intolerant to TKIs.

## Materials and methods

### Cells

The study was conducted in accordance with the principles of the Declaration of Helsinki and was approved by the Ethics Committee of the Tokai University School of Medicine (number 20I-34). Written informed consent was obtained from all patients with BCR/ABL^+^ leukemia and umbilical cord blood (CB) donors prior to sample collection. BMMNCs were isolated from the samples using Lymphoprep (StemCell Technologies) density gradient centrifugation. The CB-CD34^+^ cell fraction was prepared using the CD34 Progenitor Cell Isolation Kit (Miltenyi Biotec). BMMNCs from patients with leukemia and CB-CD34^+^ cells from healthy donors were frozen in a medium supplemented with dimethyl sulfoxide (DMSO) and fetal calf serum using a step-down freezing procedure, and stored in liquid nitrogen. Aliquots of frozen samples were thawed immediately before use. The interleukin-3-dependent mouse hematopoietic cell line, 32D, and human leukemic cell lines—K562 (blast crisis of chronic myeloid leukemia), KCL22 (Philadelphia chromosome-positive human acute lymphoblastic leukemia), HL60 (promyelocytic leukemia), Jurkat (T-cell lymphoblastic leukemia), and Raji (B-cell lymphoblastic leukemia)—were purchased from the American Type Culture Collection. The MOLM-14 (human monocytic leukemia) cell line was kindly provided by the Cell Biology Institute, Research Center, Hayashibara Biochemical Laboratories. K562 cells stably expressing luciferase (Luc-K562 cells) were obtained by transduction of lentivirus containing the luciferase-enhanced green fluorescent protein gene (CSII-CMV-Luciferase2-EGFP). The 32D-p210^BCR/ABL^ and T315I-32D-p210^BCR/ABL^ cell lines were established by infection of 32D cells with MSCV*-BCR-ABL-*ires-*GFP* and MSCV-*BCR-ABL1-T315I* mutant-ires-*GFP*, respectively, as previously described^51^. The cells were maintained in RPMI 1640 medium supplemented with 10% (20% for KCL22 cells) heat-inactivated fetal bovine serum and antibiotics (100 U penicillin/mL and 100 μg streptomycin/mL) at 37 °C in a humidified 5% CO_2_ atmosphere.

### Reagents

Pterostilbene was kindly provided by the Molecular Physiological Chemistry Laboratory (Tokyo, Japan), dissolved in DMSO to yield a 100 mM stock solution, and stored at − 20 °C. The TKI dasatinib, the pan-caspase inhibitor z-VAD, and the MEK inhibitor U0126 were purchased from Cayman Chemical, Peptide Institute, and Selleck Chemicals, respectively.

### Cell culture

Reagents were added at the indicated concentrations to 96-well culture plates containing 1 × 10^4^ cells/well. Unless otherwise specified, the concentrations of pterostilbene, dasatinib, z-VAD, and U0126 were 200 μM, 20 nM, 30 mM, and 10 μM, respectively. The reagents in the treatment groups were diluted with RPMI-1640, and the corresponding volumes of DMSO and RMPI-1640 were added to the vehicle control group. When pterostilbene was added to the cell culture media along with z-VAD or U0126, pterostilbene was added 2 or 3 h after addition of the other inhibitors. Unless otherwise specified, 20 h after the addition of pterostilbene, the cells were harvested.

### Measurement of apoptosis

The cells were harvested and stained with allophycocyanin-labeled annexin V (BD Biosciences) and propidium iodide (PI) (Roche), according to the manufacturer’s instructions. The treated cells were then analyzed using a FACScan flow cytometer (Becton Dickinson). The annexin V^+^/PI^+^ cell fraction indicates late apoptotic cells.

### Capillary western immunoassay

Cell pellets were suspended in 0.1 mL of ice-cold RIPA buffer (Invitrogen) and incubated on ice for 1 h. Protein concentrations were determined using a DC protein assay (Bio-Rad). Protein expression was evaluated by capillary western immunoassay using a Wes machine (ProteinSimple), according to the manufacturer’s instructions. This system automates protein loading, separation, immunoprobing, washing, and detection, and allows absolute protein quantitation. Briefly, samples were mixed with Simple Western Sample Buffer and standards to a final concentration of 1 μg/μL, and were then reduced and denatured. The prepared samples, primary and secondary antibodies, and chemiluminescent substrate were dispensed in microliter volumes into the designated wells in an assay plate. The prepared assay plate and a capillary cartridge were loaded into the Wes machine, and the solutions were run through the capillaries. Proteins were separated in the capillaries as they migrated through a stacking and separation matrix. The separated proteins were immobilized on the capillary wall via a proprietary, photoactivated capture chemical reaction. Target proteins were then identified with a primary antibody, and subsequent immunodetection using a horseradish peroxidase-conjugated secondary antibody and chemiluminescent substrate. The chemiluminescence reactions were measured and digital blot images were captured. Antibodies against c-ABL, phospho-c-ABL, ABL, phospho-AKT, NF-κB p65, phospho-NF-κB p65, caspase-3, cleaved caspase-3, caspase-9, cleaved caspase-9, ubiquitin, p27, ERK, phospho-ERK, PERK, phospho-PERK, and phospho-eIF2α were purchased from Cell Signaling Technology. Antibodies against eIF2α and CHOP were purchased from Santa Cruz Biotechnology. Antibodies against ATF6 and ATF6 p50 were purchased from Novus Biologicals. The antibody against glyceraldehyde-3-phosphate dehydrogenase (GAPDH) was purchased from Sigma-Aldrich.

### Reverse transcription quantitative polymerase chain reaction (PCR)

RNA was isolated using the RNeasy Mini Kit (Qiagen) and was reverse transcribed. Each target cDNA was amplified via PCR on the same plate using the TaqMan Gene Expression Assays (Thermo Fisher Scientific) and the ABI 7300 Real-Time PCR System (Thermo Fisher Scientific). The TaqMan probe used was derived from BCR/ABL (Thermo Fisher Scientific; Assay ID: Hs03024541). The relative amounts of the target genes were determined with reference to *18S* rRNA. Comparative threshold cycle (C_T_) analysis was used to quantify transcripts. The values were calculated using the 2^−ΔΔCT^ method.

### Transplantation and treatment procedures

Eight-week-old female immunodeficient SHO mice were purchased from CLEA Japan. A mixture of 2 × 10^6^ Luc-K562 cells or T315I-32D-p210^BCR/ABL^ cells and basement membrane matrix (BD Biosciences) was subcutaneously injected into the right flank of the mice. Day 8 after transplantation, the mice were administered vehicle, dasatinib (10 mg/kg/day), or pterostilbene (25 mg/kg/day) orally for a total of 14 times in 18 days. The tumor volume was measured with calipers at the times indicated in Fig. 5a,c. The general toxicity of the reagents was measured during the experiments. All experimental procedures and protocols involving animals were reviewed and approved by the Animal Care Committee of the Tokai University and were in compliance with the ARRIVE guidelines^52,53^, and all methods were carried out in accordance with relevant guidelines and regulations.

### Bioluminescence imaging

Bioluminescence imaging was performed with a highly sensitive, cooled CCD camera mounted in a light-tight specimen box (in vivo imaging system [IVIS]; Xenogen Corporation), as described previously^54^. Briefly, for in vivo imaging, mice transplanted with Luc-K562 cells were injected with D-luciferin (150 mg/kg). The light emitted from the mice was detected using the IVIS camera, integrated, digitized, and displayed. The total flux of photons on the images, which correlated well with tumor volume, was estimated by region of interest (ROI) measurements, which converted the surface radiance (photons/s/cm^2^/sr) to the total flux of photons (photons/s) using the Living Image Software (Caliper Life Sciences).

### Statistics

All experimental results were expressed as the arithmetic mean and standard deviation values. Student’s *t*-test was used to evaluate the statistical significance of the differences between unpaired groups. Statistical significance was set at *P* < 0.05.

## Supplementary Information

Supplementary Information.
