# Supplementary material for: Pterostilbene downregulates BCR/ABL and induces apoptosis of T315I-mutated BCR/ABL-positive leukemic cells
Source: Sci Rep. 2022 Jan 13;12:704. doi: 10.1038/s41598-021-04654-1 (PMC8758722; doi:10.1038/s41598-021-04654-1)

Supplementary Information for:

Pterostilbene downregulates BCR/ABL and induces apoptosis of T315I-mutated BCR/ABL-positive leukemic cells

Shohei Kawakami[1,2], Mitsuyo Tsuma-Kaneko[2], Masakazu Sawanobori[1], Tomoko Uno[2], Yoshihiko Nakamura[2], Hideyuki Matsuzawa[3], Rikio Suzuki[1,2], Makoto Onizuka[1,2], Takashi Yahata[2,4], Kazuhito Naka[5]、Kiyoshi Ando[1,2], Hiroshi Kawada[1,2]

[1]Division of Hematology/Oncology, Department of Internal Medicine, Tokai University School of Medicine, 143 Shimokasuya, Isehara, Kanagawa 259-1143, Japan, [2]Research Center for Regenerative Medicine, Tokai University School of Medicine, 143 Shimokasuya, Isehara, Kanagawa 259-1143, Japan, [3]Support Center for Medical Research and Education, Tokai University School of Medicine, 143 Shimokasuya, Isehara, Kanagawa 259-1143, Japan, [4]Department of Innovative Medical Science, Tokai University School of Medicine, Tokai University School of Medicine, 143 Shimokasuya, Isehara, Kanagawa 259-1143, Japan, [5]Department of Stem Cell Biology, Research Institute for Radiation Biology and Medicine, Hiroshima University, 1-2-3 Kasumi, Minami-ku, Hiroshima, 734-8553, Japan.

Corresponding author: Hiroshi Kawada, M.D., Ph.D.
Division of Hematology/Oncology, Department of Medicine, Tokai University School of Medicine, 143 Shimokasuya, Isehara, Kanagawa 259-1143, Japan.
Phone: +81-463-93-1121 ext. 2232, e-mail: kawada@tokai.ac.jp

Supplementary Figure 1. Time-course changes in the flow cytometric profiles of leukemic cells cultured in the presence of pterostilbene

T315I-32D-p210$^{BCR/ABL}$ cells were cultured in the presence of pterostilbene, collected at the indicated time points, and subjected to flow cytometric analysis. The annexin V^{+}/PI^{+} cell fraction resulted from the increasing numbers of cells first becoming annexin V^{+}/PI^{-} and then double-positive, indicating that most cells underwent apoptosis.

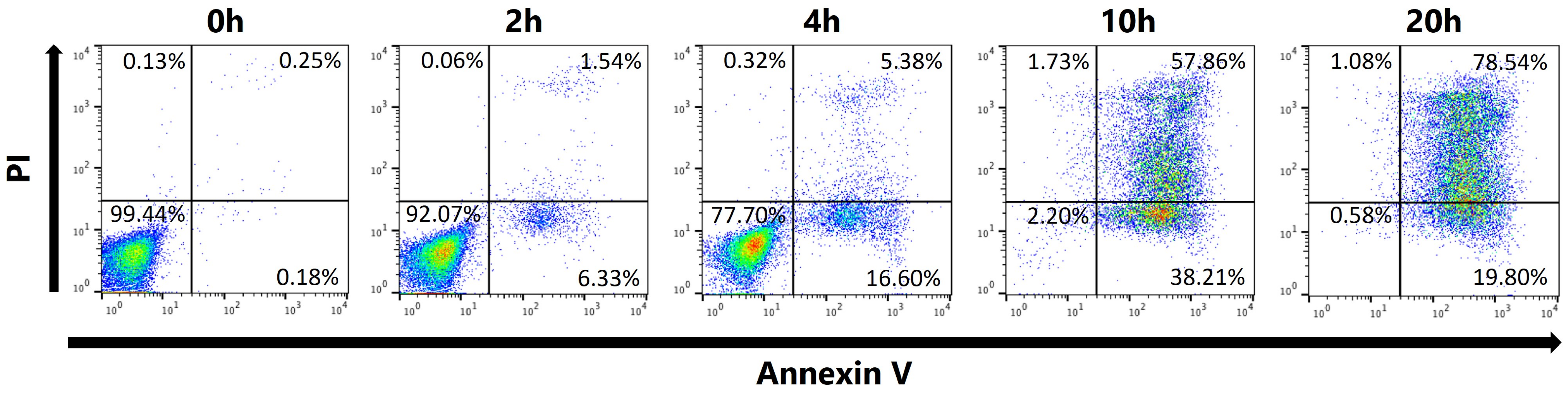

Supplementary Figure 2. Time-course changes in percentages of annexin V⁺/PI⁺ cells cultured in the presence of pterostilbene

T315I-32D-p210BCR/ABL cells were cultured in the presence of pterostilbene, collected at the indicated time points, and subjected to flow cytometric analysis. *P<0.05 compared with 20 h incubation.

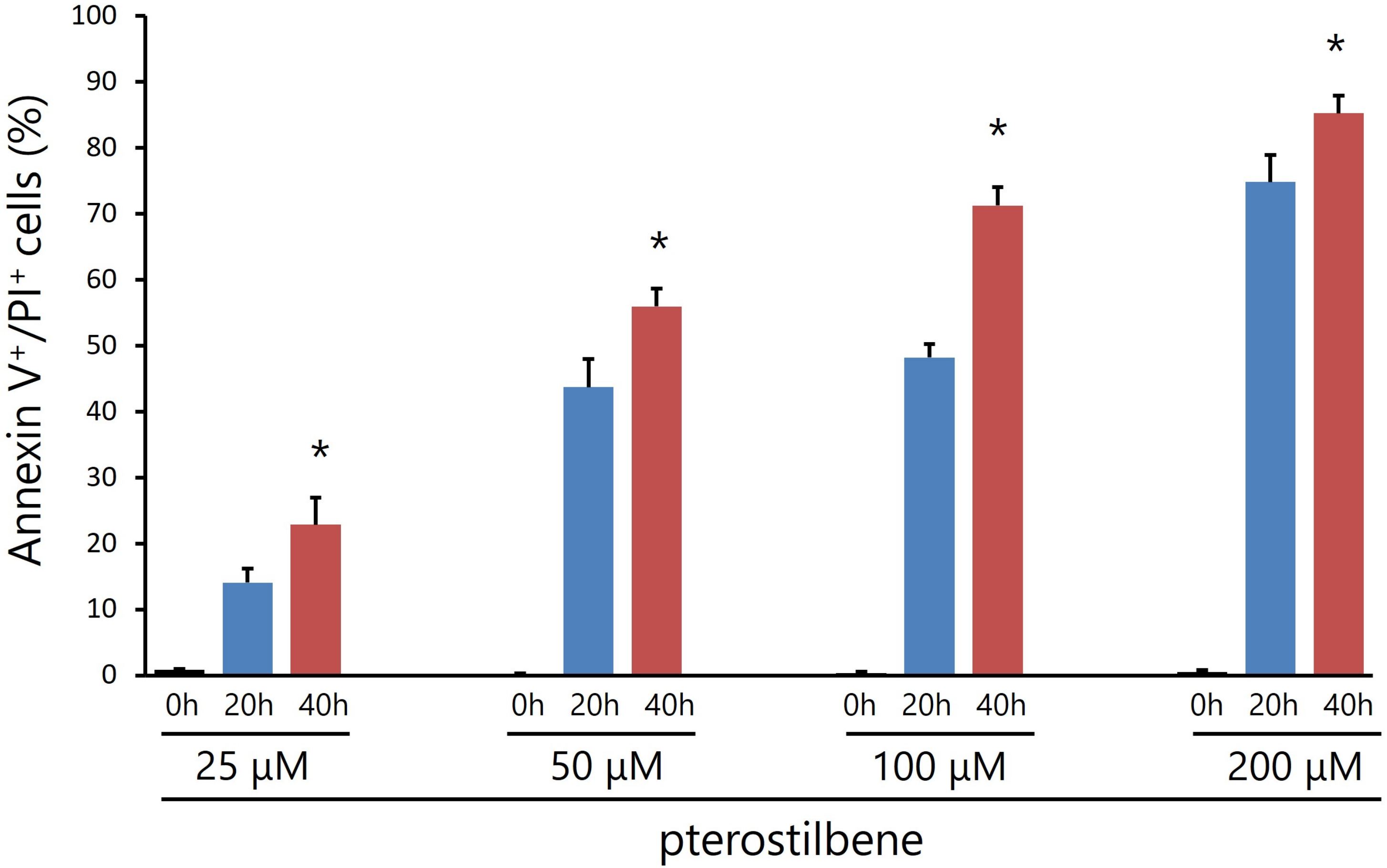

Supplementary Figure 3. Uncropped images of the blots presented in the main figure 2a.

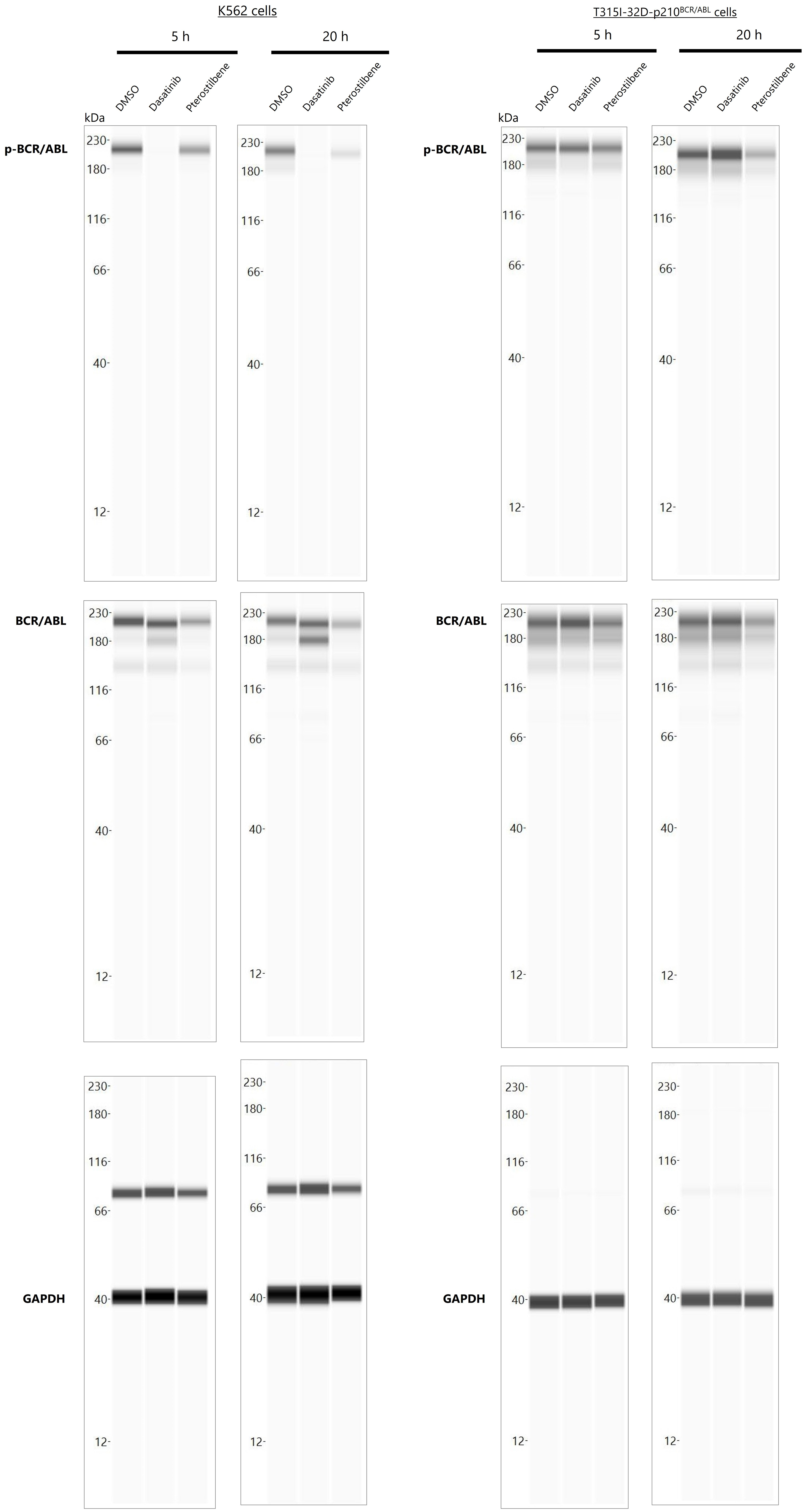

Supplementary Figure 4. Uncropped images of the blots presented in the main figure 2c.

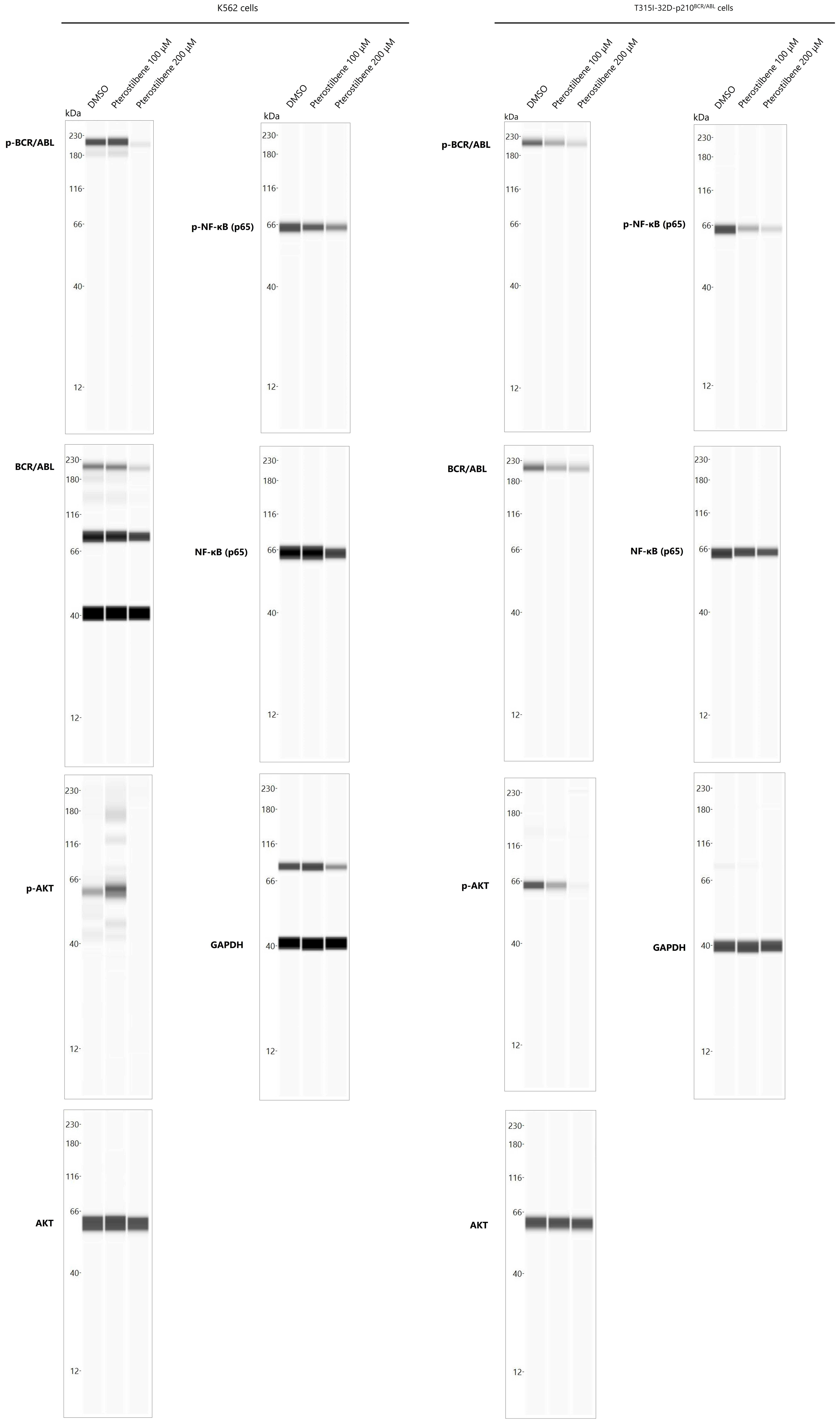

Supplementary Figure 5. Uncropped images of the blots presented in the main figure 3a.

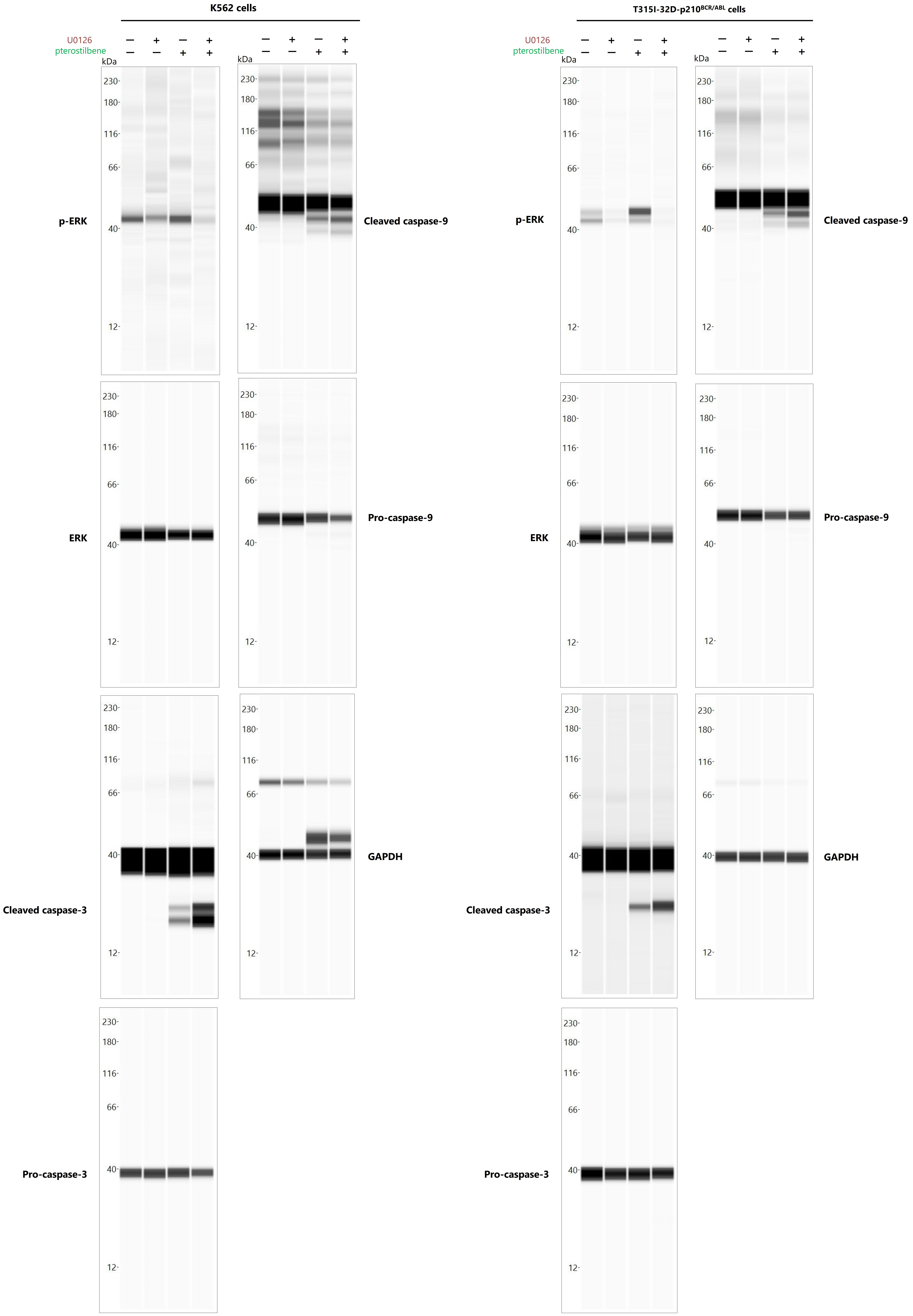

Supplementary Figure 6. Uncropped images of the blots presented in the main figure 4a.

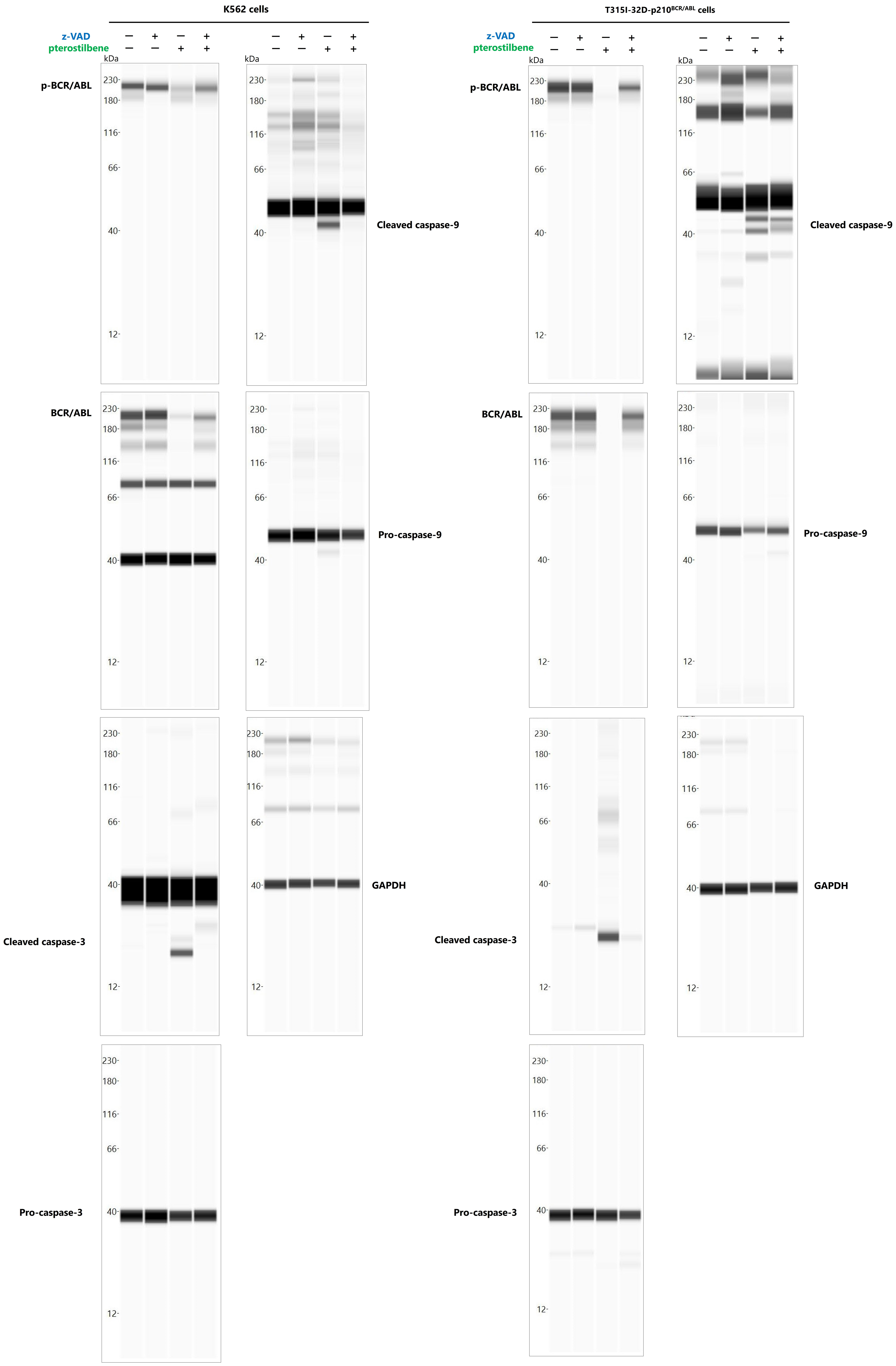

Supplementary Figure 7. Uncropped images of the blots presented in the main figure 4c.

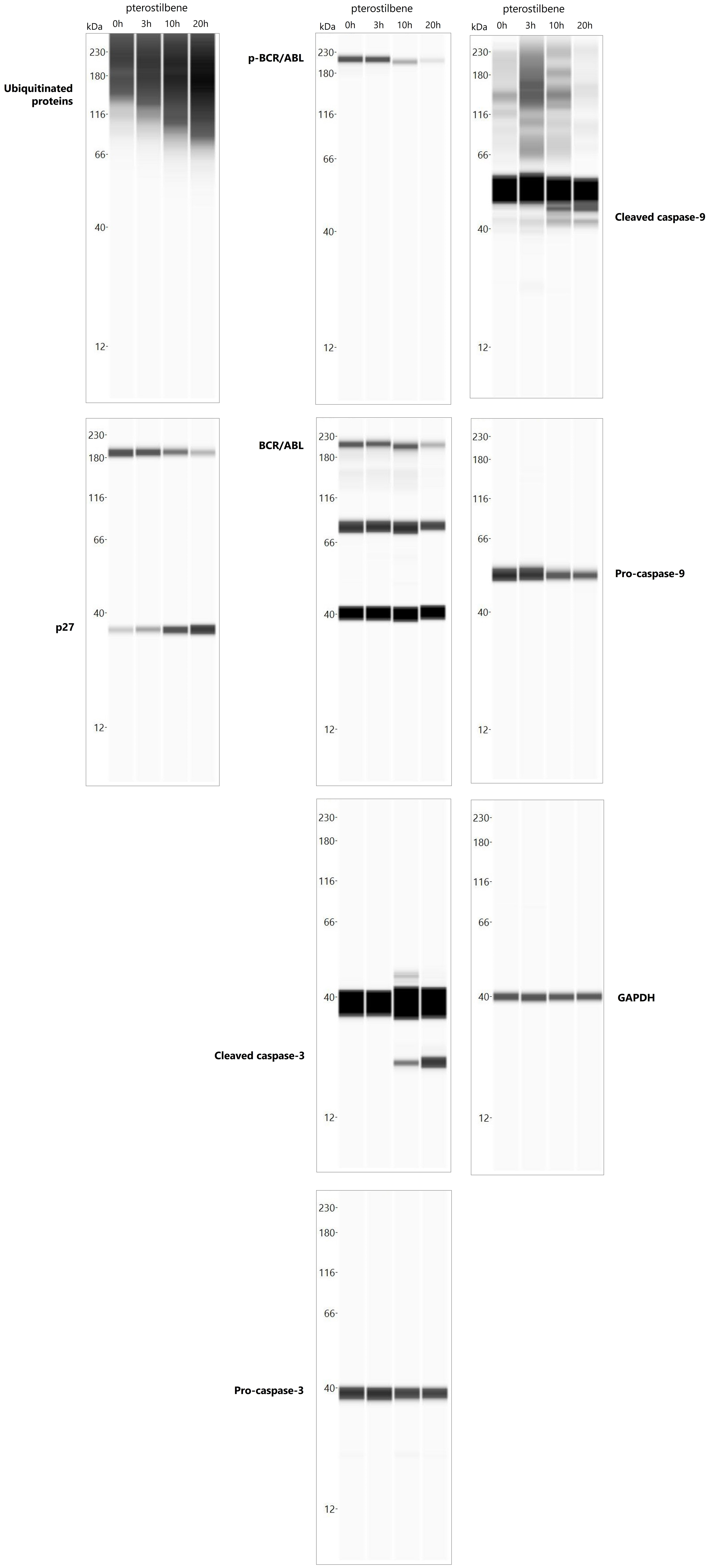

Supplementary Figure 8. Uncropped images of the blots presented in the main figure 4d.

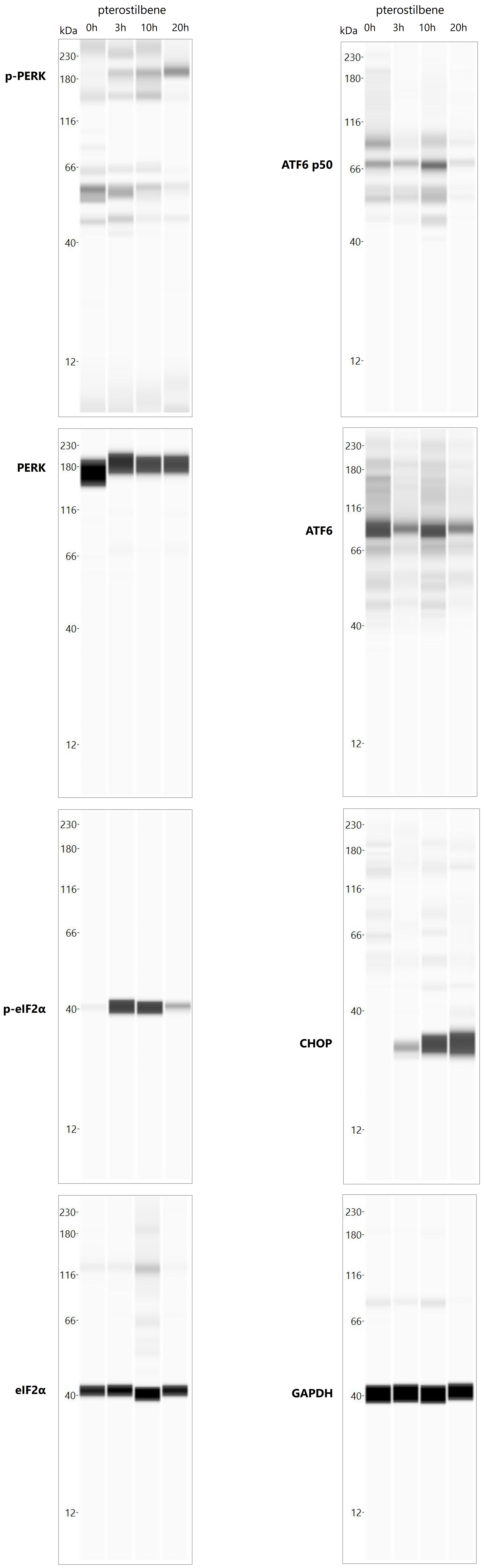

Supplement: Supplementary file 1 — Supplementary Information. [file 41598_2021_4654_MOESM1_ESM.pdf]
